# Structure and dynamics of supercooled water in the hydration layer of poly(ethylene glycol)

**DOI:** 10.1063/4.0000158

**Published:** 2022-09-08

**Authors:** Yuqing Li, Zehua Han, Changli Ma, Liang Hong, Yanwei Ding, Ye Chen, Junpeng Zhao, Dong Liu, Guangai Sun, Taisen Zuo, He Cheng, Charles C. Han

**Affiliations:** 1 Institute of High Energy Physics (IHEP), Chinese Academy of Sciences (CAS), Beijing 100049, China; 2 Spallation Neutron Source Science Center, Dongguan 523803, China; 3 University of Chinese Academy of Science, Beijing 100049, China; 4 School of Physics and Astronomy, Shanghai Jiao Tong University, Shanghai 200240, China; 5 Department of Chemical Physics, University of Science and Technology of China, Hefei 230026, China; 6 Faculty of Materials Science and Engineering, South China University of Technology, Guangzhou 510640, China; 7 Key Laboratory of Neutron Physics, Institute of Nuclear Physics and Chemistry, China Academy of Engineering Physics, Mianyang 621999, China; 8 Institute for Advanced Study, Shenzhen University, Shenzhen 508060, China

## Abstract

The statics and dynamics of supercooled water in the hydration layer of poly(ethylene glycol) (PEG) were studied by a combination of quasi-elastic neutron scattering (QENS) and molecular dynamics (MD) simulations. Two samples, that is, hydrogenated PEG/deuterated water (h-PEG/D_2_O) and fully deuterated PEG/hydrogenated water (d-PEG/H_2_O) with the same molar ratio of ethylene glycol (EG) monomer to water, 1:1, are compared. The QENS data of h-PEG/D_2_O show the dynamics of PEG, and that of d-PEG/H_2_O reveals the motion of water. The temperature-dependent elastic scattering intensity of both samples has shown transitions at supercooled temperature, and these transition temperatures depend on the energy resolution of the instruments. Therefore, neither one is a phase transition, but undergoes dynamic process. The dynamic of water can be described as an Arrhenius to super-Arrhenius transition, and it reveals the hydrogen bonding network relaxation of hydration water around PEG at supercooled temperature. Since the PEG-water hydrogen bond structural relaxation time from MD is in good agreement with the average relaxation time from QENS (d-PEG/H_2_O), MD may further reveal the atomic pictures of the supercooled hydration water. It shows that hydration water molecules form a series of pools around the hydrophilic oxygen atom of PEG. At supercooled temperature, they have a more bond ordered structure than bulk water, proceed a trapping sites diffusion on the PEG surface, and facilitate the structural relaxation of PEG backbone.

## I. INTRODUCTION

The functional activity of biomacromolecules is closely related to its hydration water. At extremely low temperature, they have no conformation flexibility and show no biological functions. Both hydration water and biomacromolecules have no flexibility. Signs of life cannot be found. They cannot regain their flexibility with a soft conformation until it increases to about 200 K,^1^ regardless of their molecular types. It is generally believed that the existence of hydration water governs this process. Thus, the storage temperature of most biological organ refrigerators should be below 200 K. For example, the Pfizer/BioNTech COVID-19 vaccine requires storage at 193 K with a shelf life up to six months.^2^

In light of this, scientists have made all their efforts to elucidate the relationship between hydration water and biomacromolecules. It was reported that protein will lose its enzyme activity when water is less than 0.2 g for per g protein. The enzyme activity shows a rapidly increasing when water increases from 0.2 to 0.5 g/g^3^ in which 0.3 g/g is sufficient to cover the protein surface with a monolayer of water to fully activate its function.^4^ Frauenfelder *et al.* used a solvent-slaved mechanism to explain the transition at ∼200 K; that is, the motion of biomacromolecules is controlled by the motion of its hydration shell.^5,6^ Moreover, the following researchers argued that it is a more complicated process. Wood *et al.* found that the membrane protein may be less strongly coupled to its hydration water than soluble proteins.^7^ Khodadadi *et al.* found that the dynamics of hydrated proteins, RNA, and DNA display distinct temperature dependence.^8^ Russo *et al.* showed that hydrophobic and hydrophilic interfaces have different influence on the activation of water diffusion.^9^ Weik *et al.* proposed that nanosecond-scale membrane dynamics are insensitive to the state of the water at low temperature.^10^ Thus, nature of this transition is still vague.

So far, a phenomenal “fragile-to-strong crossover” (FSC) of hydration water is widely used to describe this process.^11^ Properties of “fragile” and “strong” depend on how the liquid dynamical properties changed with temperature, and FSC of water is a crossover from super-Arrhenius at high temperature to Arrhenius at low temperature^12^

$$\text{Arrhenius}\;\;\;\tau =\tau_{0}\;\text{exp}\;(\frac{E_{A}}{k_{B}T}),$$ (1)

$$\text{Super}-\text{Arrhenius}:\;\tau =\tau_{0}\;\text{exp}\;(\frac{KT_{0}}{T-T_{0}}).$$ (2)

Here, $E_{A}$ represents activation energy, $T_{0}$ is the relaxation time divergence temperature, and K is a constant providing the measure of fragility. The debate on whether it is a phase transition continues. Chen *et al.* suggested that a structural phase transition from a predominantly low-density liquid (LDL) to high-density liquid (HDL) form of hydration water should take place at FSC (220 K).^13,14^ While Khodadadi *et al.* thought that this transition is caused by the resolution window of neutron spectrometer,^15^ Li *et al.* found that the mean-squared displacements (MSDs) of dry protein also has a transition at ∼200 K, and they proposed that this transition is a dynamic process and may origin from the collective heavy atom motions of protein.^1,16^

Because of the significant differences in incoherent scattering length between hydrogen (25.28 Fermi) and deuterium (4.04 Fermi), quasi-elastic neutron scattering (QENS) can provide the most straightforward way to assess the dynamics of macromolecule and its hydration water, separately. QENS can reveal the displacement of atoms or molecules over distances from angstroms to a few tens of nanometers with the time scale from picoseconds (ps) to nanoseconds (ns). In order to fully take the advantages of QENS, we use poly(ethylene glycol) (PEG) as the model water-soluble macromolecule. On the one hand, it is the simplest water-soluble macromolecule. Its oxygen–oxygen pair distribution function is similar to water, so it is existent as solute, which will not change the structure of hydration water. On the other hand, hydrogenated and deuterated PEG with similar molecular weight and molecular weight distribution is easily synthesized by anionic ring-opening polymerization (ROP). No exchange of hydrogen or deuterium atoms occurs between the solute and the solvent. Dynamics of h-PEG/D_2_O and d-PEG/H_2_O can be compared directly.

In this manuscript, QENS and molecular dynamics (MD) simulations are brought together to reveal the time-resolved atomic pictures of supercooled water in the PEG hydration layer. h-PEG/D_2_O and d-PEG/H_2_O with the same molar ratio of ethylene glycol (EG) to water are compared to separate the dynamics of PEG and water, and different QENS instruments with different energy resolutions are used to show the nature of fragile-to-strong dynamic crossover (FCS). Since MD simulation results are comparable with the characteristic relaxation time of hydration water by QENS, we believe that it can also reveal the temperature-dependent atomic pictures. Water molecules around PEG form a lot of pools. At 150 K, both PEG and its hydration water are frozen. When it increases to ∼209 K, the collective motion of hydration water network in pools is possible. This movement enables the cranking motion of CH_2_ in PEG's backbone. When it comes to ∼220 K, the jump diffusion of hydration water between different pools happens and facilitates the structural relaxation of PEG at ∼234 K.

## II. EXPERIMENTAL SECTION

All chemicals were purchased from Aldrich except deuterated ethylene oxide (d-EO, C_2_D_4_O), which was purchased from Cambridge Isotopes. h-PEG and d-PEG were synthesized by anionic ROP of h-EO and d-EO with water and heavy water as the initiator, respectively, and a metal-free Lewis pair consists of a phosphazene base and triethylborane as the catalyst.^17^ The number average molecular weight and molecular weight distribution of h-PEG (d-PEG) were determined by size exclusion chromatography [N,N-dimethyl-Formamide (DMF)], 50 °C, PEG standards) to be 2500 (2700) g/mol and 1.06 (1.08).

### A. Small-angle neutron scattering

Small-angle neutron scattering (SANS) measurements were performed on the SANS instrument Suanni at China Mianyang Research Reactor (CMRR), and all the experiments were carried out at 20 °C.^18^ The selected neutron wavelength was 5.3 Å, with wavelength spread (Δλ/λ) around 18%. Two sample-to-detector distances, L_2_ = 2.5 and 10.44 m, were used. The measured scattering vector, q, was from 0.002 to 0.13 Å^−1^. Background from the dark current and the empty cell were subtracted. Igor Pro software was used to fit the data.^19^

### B. Differential scanning calorimetry

Differential scanning calorimeter (DSC) measurements were performed on PerkinElmer DSC8000. Samples weighted about 12 mg were put in aluminum containers for measurements. Indium was used for temperature and enthalpy calibration. Samples were then cooled down from 293 K to about 123 K rapidly with liquid nitrogen and held at 123 K for 3 min before performing the subsequent heating procedure. The cooling and heating rate was 10 K/min. The transition temperatures were extracted by analyzing the inflection from the onset point of the steps in the cooling and heating curves.

### C. Quasi-elastic neutron scattering

Two QENS spectrometers were utilized to elucidate the temperature dependence relaxations over a wide energy range. High-flux backscattering spectrometer (HFBS)^20^ NG2 at NIST (the National Institute of Standards and Technology) was used for elastic scans in the energy range ∼17 *μ*eV with a resolution ∼1 *μ*eV and q range 0.25–1.75 Å^−1^. The high-resolution inelastic spectrometer (IRIS) at ISIS (Rutherford-Appleton Laboratory, Harwell Science and Innovation Campus, UK) was used in the energy range from 17 *μ*eV to 1 meV with a resolution 17.5 *μ*eV and q range 0.53–1.85 Å^−1^ for both elastic scans and quasi-elastic measurements.^21^ Elastic scans were performed with a heating rate of 1 K/min from 10 K to room temperature. Combination of these two spectrometers covered a broad energy range, which was large enough to measure dynamics faster than ps up to ∼1 ns. In addition, QENS experiments were performed on IRIS for d-PEG/H_2_O samples at 260, 278, 298, 308, and 318 K, respectively, with a measurement time of 4 h at each temperature. A vanadium standard was measured at 10 K for the resolution calibration.

### D. Molecular dynamics simulations

MD were performed with the GROMACS (version 4.8.5) package with an all-atom OPLS force field (OPLS-AA).^22^ We randomly put 55 OPLS-AA PEG chains with 20 monomers together with 1165 TIP4P water molecules (water mass fraction is 0.3) in a cubic box with periodic boundary conditions. After an energy minimization of 5000 steps with the steepest descent algorithm, the system got equilibrium in the condition of constant-pressure and constant-temperature (NPT) for 10 ns at 373 K (Berendsen thermostat and Parrinello–Rahman barostat are used for isobaric–isothermal control). Then, eight simulations at different temperatures (300, 280, 260, 240, 220, 200, 180, and 150 K) were performed, starting each simulation from the final configuration of the closest temperature. The simulations in a pure water box with 1165 TIP4P water molecules were also performed at 220 K. All the simulations were conducted at a constant pressure of 1 bar. The Lennard–Jones interactions were truncated at 1.4 nm in PEG hydration system and at 1.0 nm in the pure water system. Long-range Coulomb interaction was computed using the particle-mesh Ewald (PME) method with 0.12 nm spacing of the grid points in reciprocal space, and the electrostatic interactions calculated with the PME method were truncated at 0.9 nm. All bonds were constrained at their equilibrium values using the linear constraint solver algorithm (LINCS24). All simulations used for statistical analyses have simulation time ranging from 50 ns for high temperatures to 1 *μ*s for the lowest temperature.

## III. RESULTS AND DISCUSSION

### A. Calibration of PEG

To prove that the deuteration effects on chain conformation and interactions can be ignored at room temperature, zero average contrast matching (ZAC) must be done first.^23^ For h-PEG/d-PEG/H_2_O/D_2_O mixture, the scattering intensity is given by the following equation:

$$\frac{d\Sigma }{d\Omega }={\left(\rho_{\mathrm{dPEG}}-\rho_{S}\right)}^{2}S_{dd}(q)+{\left(\rho_{\mathrm{hPEG}}-\rho_{S}\right)}^{2}S_{hh}(q)+2(\rho_{\mathrm{dPEG}}-\rho_{S})(\rho_{\mathrm{hPEG}}-\rho_{S})S_{hd}(q),$$ (3)

where $\rho_{\mathrm{dPEG}}$, $\rho_{\mathrm{hPEG}}$, and $\rho_{S}$ are the scattering length densities of d-PEG, h-PEG, and D_2_O/H_2_O mixed solvent, respectively; $S_{dd}(q)$, $S_{hh}(q)$, and $S_{hd}(q)$ are the partial scattering factors of d-PEG, h-PEG, and their cross-term. The volume fraction of PEG in solution is $\varphi_{\mathrm{PEG}}=\varphi_{\mathrm{dPEG}}+\varphi_{\mathrm{hPEG}}$, and the volume fraction of water is $\varphi_{S}=\varphi_{H_{2}O}+\varphi_{D_{2}O}$. Since d-PEG and h-PEG have the similar molecular weight and molecular weight distribution, $S_{hh}(q)=S_{hh}(q)=-S_{hd}(q)$, Eq. (3) can be simplified to the following equation:

$$\begin{array}{c} \frac{d\Sigma }{d\Omega }={\left(\rho_{\mathrm{dPEG}}-\rho_{S}\right)}^{2}\frac{\varphi_{\mathrm{dPEG}}\varphi_{\mathrm{hPEG}}}{\varphi_{\mathrm{PEG}}{}^{2}}n_{\mathrm{PEG}}v_{\mathrm{PEG}}P_{S}(q)\\ +{\left(\rho_{\mathrm{dPEG}}\frac{\varphi_{\mathrm{dPEG}}}{\varphi_{\mathrm{PEG}}}+\rho_{\mathrm{hPEG}}\frac{\varphi_{\mathrm{hPEG}}}{\varphi_{\mathrm{PEG}}}-\rho_{S}\right)}^{2}n_{\mathrm{PEG}}\varphi_{\mathrm{PEG}}v_{\mathrm{PEG}}P_{T}(q). \end{array}$$ (4)

**FIG. 1.**
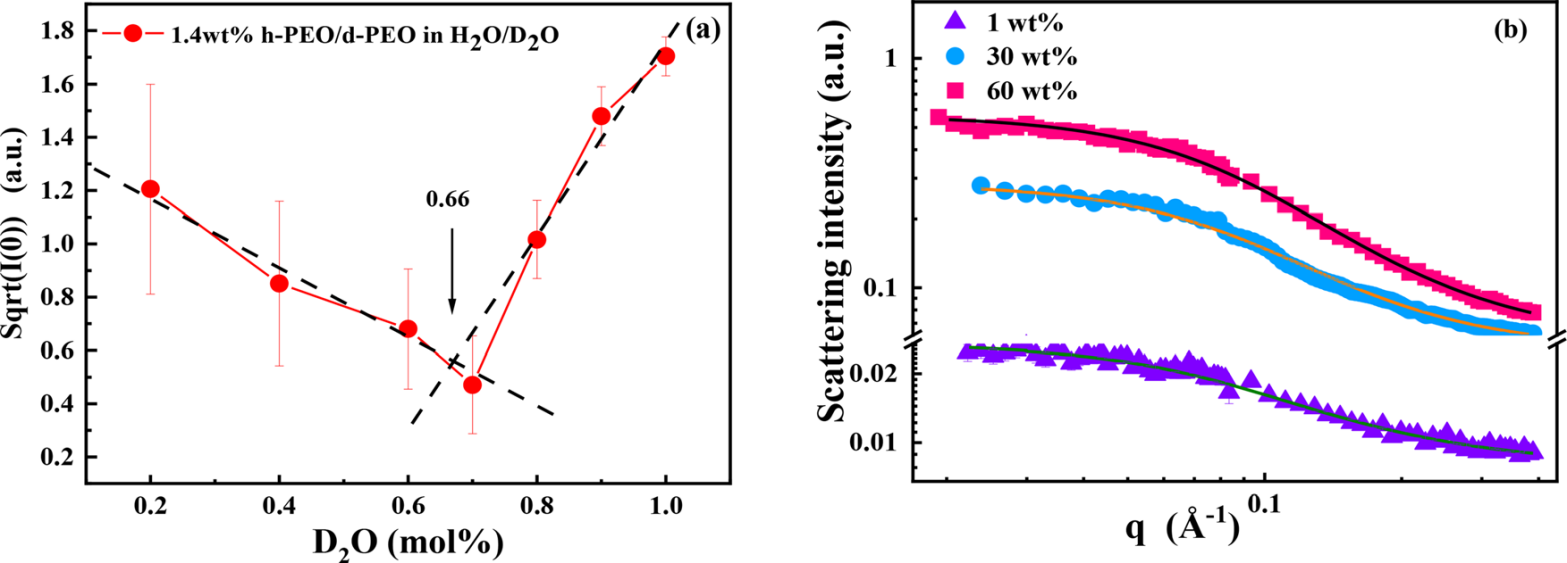
SANS scattering profiles of PEG in the H_2_O/D_2_O mixture at 20 °C. (a) SANS data of 1.4 wt. % of h-PEG/d-PEG in H_2_O/D_2_O mixture as a function of H/D ratio. The best contrast matching point is located at D_2_O: H_2_O = 0.66: 0.34 (molar ratio). (b) SANS data of 1 wt. % h-PEG/D_2_O (filled triangles), 30 wt. % h-PEG/d-PEG (molar ratio 1:1) (filled circles), and 60 wt. % h-PEG/d-PEG (molar ratio 1:1) ( filled squares) at contrast matching point.

Here, the definition of $P_{T}(Q)=P_{S}(Q)+\varphi_{\mathrm{PEG}}P_{I}$ is used. $P_{S}$ and $P_{I}$ are the form factors of single-chain and inter-chain parts. Changing the ratio of D_2_O/H_2_O, when $\rho_{\mathrm{dPEG}}\frac{\varphi_{\mathrm{dPEG}}}{\varphi_{\mathrm{PEG}}}+\rho_{\mathrm{hPEG}}\frac{\varphi_{\mathrm{hPEG}}}{\varphi_{\mathrm{PEG}}}=\rho_{S}$, the second term can be zero. Therefore, the scattered neutron intensity has a minimum with the varying of the deuterium content in the solvent. As shown in Fig. 1(a), the squared root of scattering intensity is linearly changed with the hydrogen/deuterium proportion, and the best matching point is located at D_2_O:H_2_O = 0.66:0.34 (molar ratio). This method can extract the single chain form factor without extrapolation to the zero-concentration limit.

For a polymer with excluded volume, its form factor is given by the following equation:^24–26^

$$P_{S}(q)=\frac{1}{vU^{\frac{1}{2v}}}\gamma (\frac{1}{2v},U)-\frac{1}{vU^{\frac{1}{v}}}\gamma (\frac{1}{v},U).$$ (5)

Here, γ(x,U) is the lower incomplete gamma function as the following equation:

$$\gamma (x,U)=\int_{0}^{U}dt\;\text{exp}\;(-t)t^{x-1}.$$ (6)

The variable U is given in terms of the scattering variable q as the following equation:

$$U=\frac{q^{2}R_{g}^{2}(2v+1)(2v+2)}{6}.$$ (7)

The radius of gyration has been defined as the following equation:

$$R_{g}{}^{2}=\frac{b^{2}n^{2v}}{\left(2v+1)(2v+2\right)},$$ (8)

where b is the statistical segment length, n is the degree of polymerization, and v is the excluded volume parameter. When v = 1/3, they are collapsed chains; v = 3/5 means fully swollen chains with excluded volume; and v = 1/2 are ideal Gaussian coils without free volume. The concentration-dependent conformation variation in D_2_O/H_2_O mixture (at ZAC) of PEG is shown in Fig. 1(b). It demonstrates that PEG is a Gaussian chain with excluded volume (v = 3/5) in dilute aqueous solution (1 wt. %). The excluded volume vanishes (v = 1/2) as the solution gets more crowded (30 and 60 wt. %). Thereby, Rg decreases with the increase in concentration. Because the Rg differs when polymer chains are dissolved in different good or theta solvents,^27^ we use the excluded volume parameter here to identify whether there has excluded volume or not. The fitted parameters are given in Table I. Note that 1 wt. % h-PEG in D_2_O is a dilute solution, we can measure its conformation directly. When it is higher than 30 wt. %, equal molar h-PEG and d-PEG have to be used to contrast match the 66/34 D_2_O/H_2_O solvent eliminate the second term on the right of Eq. (4).

**TABLE I.** The parameters of SANS data fitted with polymer excluded volume function.

Samples	h-PEG/D_2_O	(h-PEG/d-PEG)/(H/D water)	
PEG mass fraction	1 wt. %	30 wt. %	60 wt. %
Rg (Å)	2.3 ± 0.1	2.0 ± 0.1	1.9 ± 0.1
m^a^	1.67	2	2

^a^ m is the Porod exponent.

**FIG. 2.**
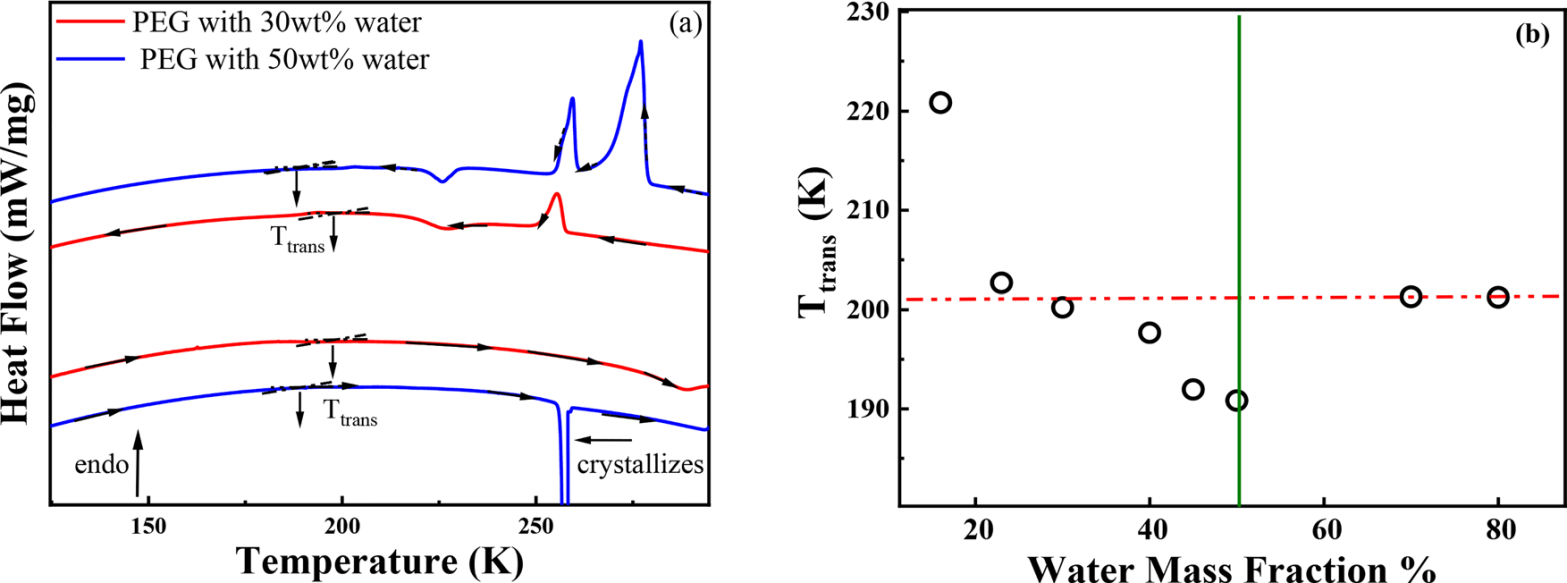
(a) The heating and cooling heat flow spectra detected by DSC. The sample consists of an equimolar mixture of d-PEG/h-PEG with 30 and 50 wt. % water. (b) Transition temperature of these samples vs water mass fraction.

Then, DSC is utilized to choose the best molar ratio of EG monomer to water. We require to keep as much hydration water as possible in QENS. In biology, water molecules in protein solution can be classified into three categories: (1) the bound internal water that occupies internal cavities and plays a structural role in the folded protein; (2) the hydration water that interacts with the solvent-exposed protein atoms and exhibits in the slow dynamics (it is also the first layer of water); (3) the bulk water that is not directly interact with the protein, but continuously exchanges with the hydration water.^13^ DSC experiment is conducted at a series of water mass fractions, that is, 16, 23, 36, 43, 49, 70, and 80 wt. %. Figure 2(a) is a typical DSC thermogram of PEG with 30 and 50 wt. % water. We noted that there is only hydration water in the 30 wt. % solution, while it also has bulk water in the 50 wt. % solution. At 50 wt. %, the bulk water crystallizes first at subzero upon cooling; then, an upshift transition of hydration water appears at ∼190 K, depending on different water contents. Upon subsequent reheating, a transition of hydration water at ∼190 K happens first, then undergoes cold crystallization at 227 K, and finally melts at 255 and 273 K (bulk water). While in 30 wt. %, the subzero crystallization peak and melting endothermic peaks disappear, and the upshift transition happened at 200 K. Therefore, the PEG/water model system has the similar thermal behavior with biomacromolecules. h-PEG and d-PEG were mixed uniformly under our experimental condition.

The water content dependence of transition temperature is shown in Fig. 2(b). The transition temperature first decreases with the increase in water mass fraction. When water mass fraction is larger than 50 wt. %, it remains unchanged. At that time, most water in the system is bulk water. They will crystallize in the cooling process. PEG with its hydration water may thus be squeezed out. The transition temperature in the reheating process is thereby independent of the original water content. Here, by making an auxiliary line from dilute solution, the cross-point is at 30 wt. % of water mass fractions. Therefore, we use this concentration in QENS experiments, corresponding to a 1:1 molar ratio of EG monomer to water. Similar DSC measurements were also conducted by Guo *et al.*, and they explained that the drop in the medium range from 30 to 50 wt. % is due to the plasticizer effect of water.^28^

### B. QENS study on the dynamics of supercooled hydration water around PEG

**FIG. 3.**
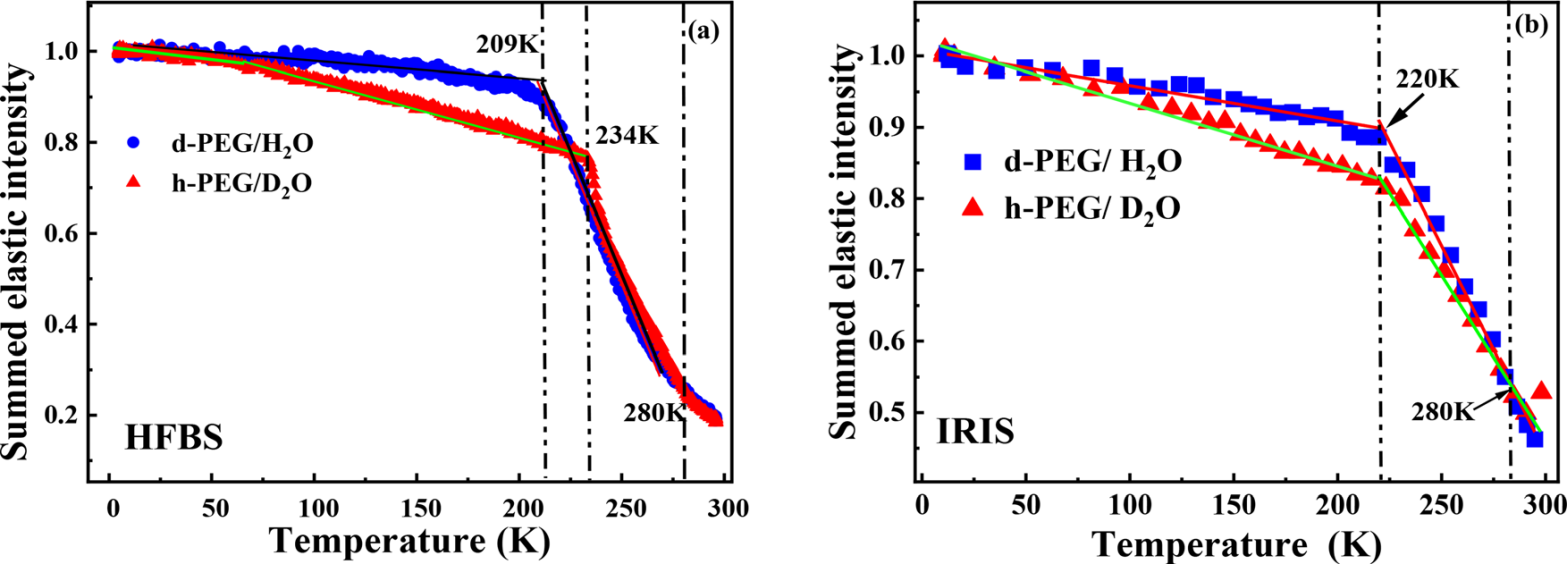
The temperature-dependent summed elastic neutron scattering of PEG and its hydration water with a molar ratio 1:1 of EG monomer to water, normalized to 10 K. (a) Data from HFBS. (b) Data from IRIS.

Now, the overall temperature-dependent dynamics of supercooled hydration water can be tracked by neutron scattering. The normalized elastic intensity is summed over a defined q range as a function of temperature for both d-PEG/H_2_O and h-PEG/D_2_O (Fig. 3). The downward bending of the summed elastic intensity curves marks a harmonic to inharmonic transition.^29^ In history, there exist two different interpretations: (i) a phase transition of hydration water;^30,31^ (ii) the temperature-dependent relaxation time reaching the limit of experimental frequency window.^15,32–34^ In a typical phase transition, molecules move together suddenly and the transition temperature with the same thermal history is necessarily independent with the instrumental resolution; while in a dynamic process, smaller atom (or group, or segment) will move first; then, the larger one follows, thereby exhibiting a transition temperature that varies with the energy resolution window. Here, we perform the elastic scattering scans with different instrumental energy resolutions. Scattering particle that moves in a time scale much lower than the characteristic time corresponding to the energy resolution is seen as an elastic scatterer, whereas a decrease in the elastic intensity can be observed for scattering particles, which move faster. This implies that a scattering particle, which moves in a time scale between the resolution time of HFBS (1 *μ*eV) and IRIS (17.5 *μ*eV), contributes as an elastic process in HFBS, and as a non-elastic process in IRIS. We noted that the dynamic process depends on its pathway. Different thermal treatments lead to different structures and dynamics. Here, the neutron elastic scans experiments were performed with a heating rate of 1 K/min from 10 K to room temperature. Therefore, they have similar thermal history and following the same temperature-changing protocol used. HFBS shows that hydration water and PEG have a transition at 209 and 234 K, respectively [Fig. 3(a)], while IRIS shows these transitions both happen at 220 K [Fig. 3(b)]. Therefore, these transitions are obviously dependent on the energy resolution of QENS. It can be concluded that the dynamics of PEG and its hydration water are uncoupled in the low energy range on the order of *μ*eV but coupled in high energy scale on the order of tens or hundreds *μ*eV. The detailed dynamic process will be discussed in the MD simulation part.

**FIG. 4.**
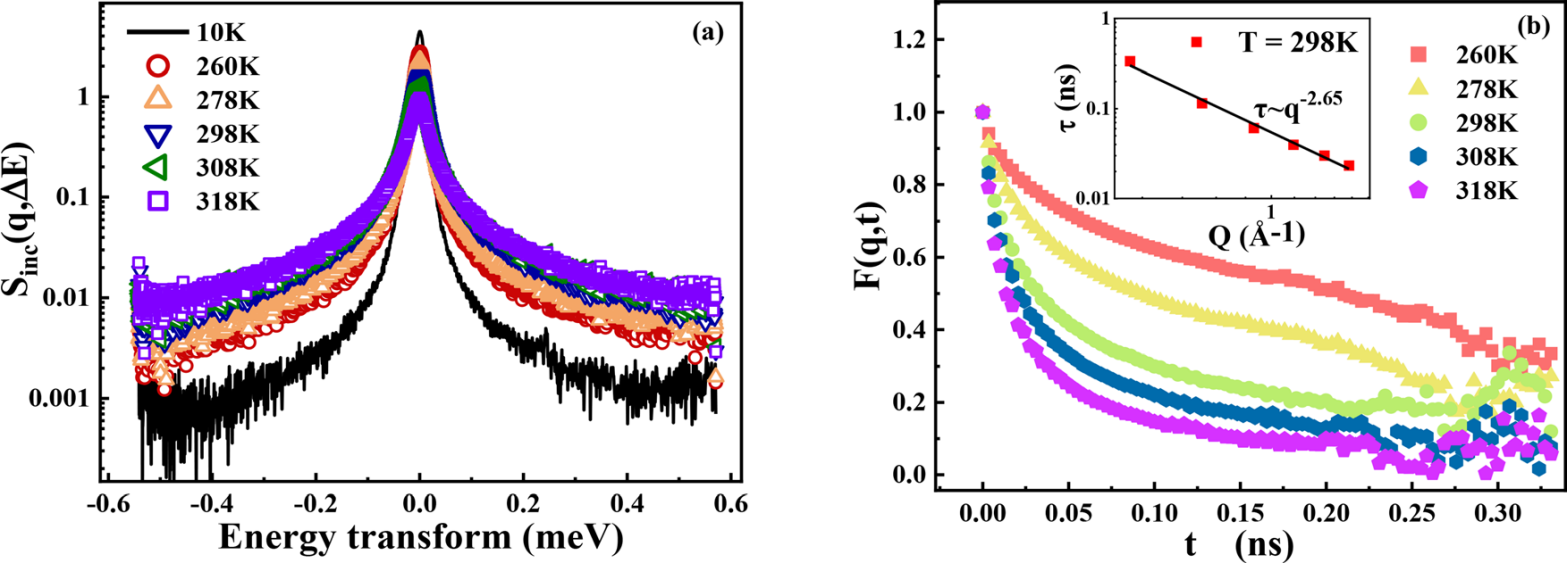
(a) Incoherent dynamic structure factor $S_{\mathrm{inc}}(q,\Delta E)$ of d-PEG/H_2_O from IRIS. To improve statistics, the spectra measured over the experimental q-window from 0.53 to 1.85 Å^−1^ are summed. For comparison, the resolution function measured at 10 K is also presented. (b) The intermediate scattering function F(q,t) of d-PEG/H_2_O is Fourier transformed from (a). The inset plot is the average relaxation time of PEG hydration water over q at 298 K.

QENS can provide information of the dynamic structure variation [Fig. 4(a)]. The incoherent dynamic structure factor can be expressed as the following equation:

$$S_{\mathrm{inc}}(q,\Delta E)=\frac{1}{2\pi }\int_{-\infty }^{+\infty }F(q,t)\;\text{exp}\;(-i\frac{\Delta E}{\hbar }t)dt.$$ (9)

Here, the intermediate scattering function (ISF) is Fourier transformed from $S_{\mathrm{inc}}(q,\Delta E)$ to the time domain. Using the relaxing-cage model (RCM),^35^ this function can be fitted by a stretching exponential function as the following equation:

$$F(q,t)=A\;\text{exp}\;[-{\left(\frac{t}{\tau }\right)}^{\beta }],$$ (10)

where τ is the average relaxation time, β is the stretching exponent, and A is the fraction of moving.^36^ We use Eq. (10) to fit F(q,t) [Fig. 4(b)]. The q-dependence of relaxation time represents the motion mode of hydration water. Li *et al.* found that at 280 K, the relaxation time of protein hydration water with hydration level of 0.4 g/g (gram H_2_O/gram protein) has a power law of 2.67 with q.^37^ The motion of water can be described as a sub-diffusion process.^38^ Here, the PEG hydration water shows a similar scaling behavior at 298 K with $\tau \propto q^{2.65}$ [Fig. 4(b), inset].

**FIG. 5.**
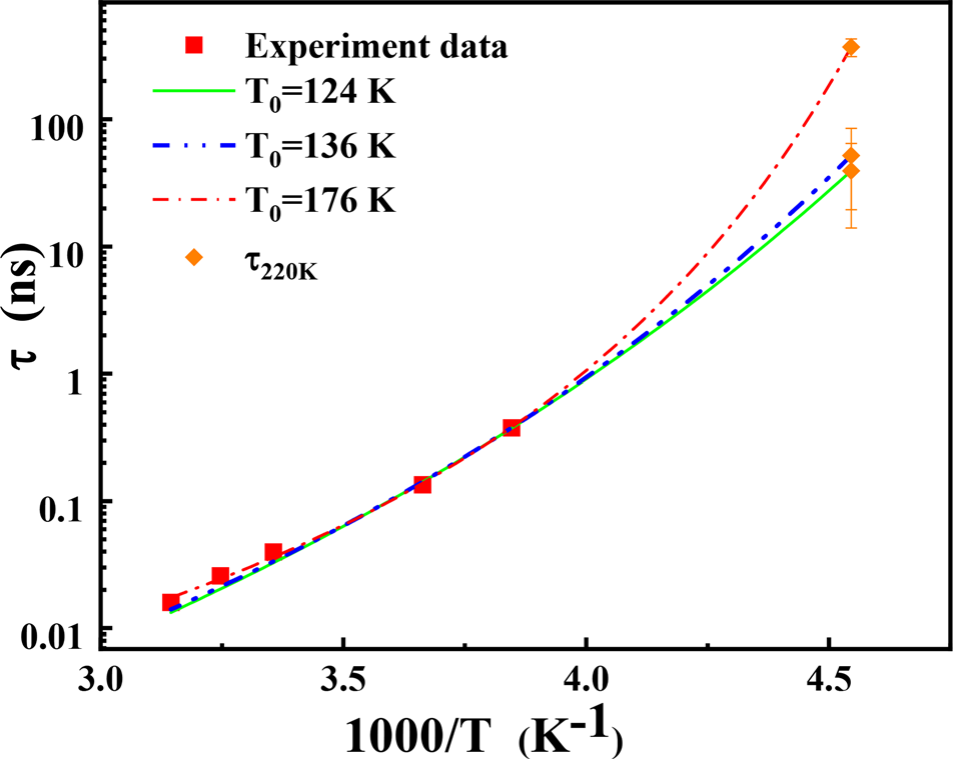
The average relaxation time of PEG hydration water (q = 1.05 Å^−1^) at various temperatures. The solid lines are the fitting lines of the super-Arrhenius function with different T_0_. The dotted lines are the extrapolation of the relaxation time to 220 K from different fitting lines.

Due to the limitation of detection energy scale of IRIS (17 *μ*eV–1 meV), it is impossible to directly measure the characteristic relaxation time of supercooled hydration water at 220 K. An extrapolation via the super-Arrhenius function from the exist data is required. According to Eq. (2), T_0_ must be chosen first. In the literature, there are three possible T_0_, that is, the glass transition temperature of hyper quenched water (T_0_ = 136 K),^39^ low-density water (LDL) (T_0_ = 124 K),^40^ and the ideal glass transition temperature of water used by Chen *et al.* (T_0_ = 176 K).^13^ Here, we have compared these three options and extrapolated the super-Arrhenius fitted curves to 220 K (the transition temperature of d-PEG/H_2_O detected by IRIS) (Fig. 5). Here, q = 1.05 Å^−1^ is the averaged q that explored experimentally. The fitted parameters are shown in Table II. The differences between the fitting results are relatively small. Given the relatively small temperature range of the measurements, it is difficult to determine with the precision fragility of hydration water.

**TABLE II.** Temperature dependence of the average relaxation time for hydration water fitted by Eq. (2) at q = 1.05 Å^−1^, and extrapolated to 220 K.

Parameters	τ_0_ (ns)	K	T_0_ (K)	τ (ns) at 220 K
$\tau =\tau_{0}\text{exp}(KT_{0}/(T-T_{0}))$	5.2 × 10^−6^ ± 2.3 × 10^−6^	12.3 ± 0.5	124	39 ± 19
	1.2 × 10^−5^ ± 4.4 × 10^−6^	9.4 ± 0.3	136	52 ± 22
	2.0 × 10^−4^ ± 2.4 × 10^−5^	3.6 ± 0.1	176	370 ± 70

### C. Time-resolved atomic picture of supercooled hydration water from MD simulations

**FIG. 6.**
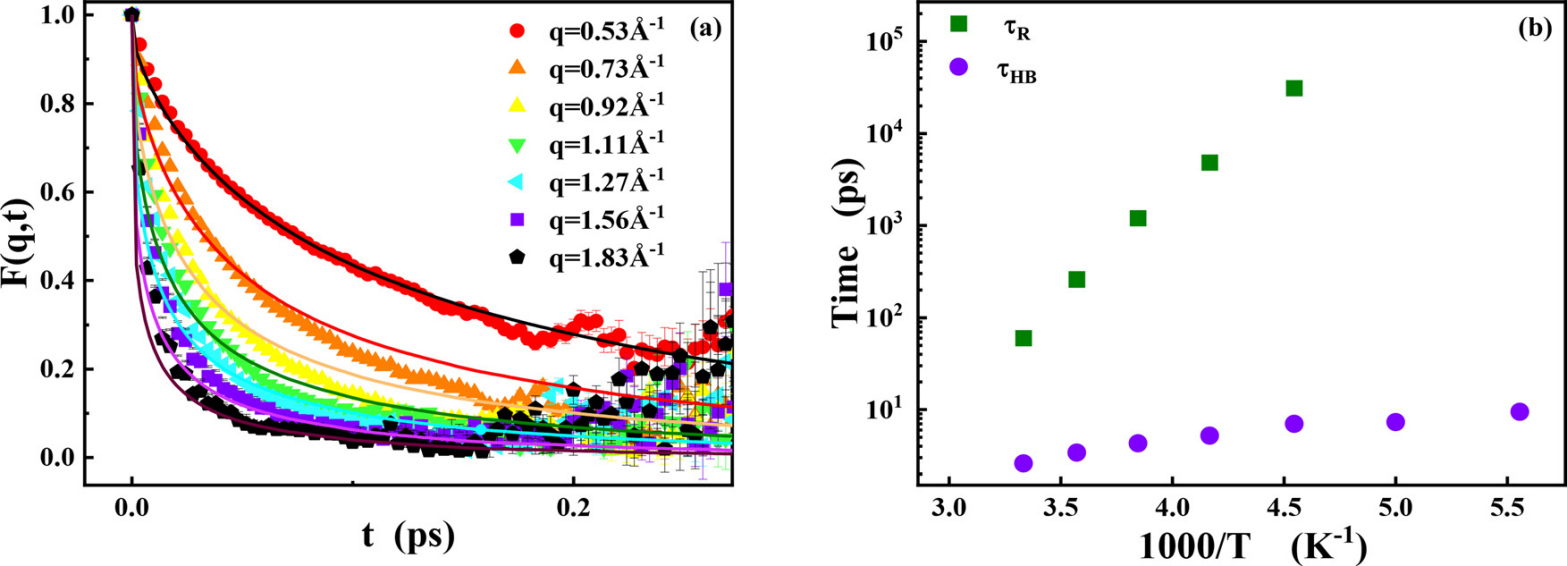
(a) Intermediate scattering function, F(q,t), at various momentum transfers obtained from experiments and MD simulations for PEG/H_2_O at 318 K. (b) Hydrogen bond lifetime and structural relaxation time between PEG and hydration water at various temperatures through two correlation functions.

The intermediate incoherent dynamic structure factors, F(q,t), at different scattering vectors obtained from both experiments and MD simulations are shown in Fig. 6(a). They can compare with each other. Therefore, we believe that the structure and dynamics of hydration water can be reproduced by simulation. Chandra have used a hydrogen bond time correlation function to characterize the time-dependent fluctuation of hydrogen bonds population.^41^ The hydrogen bonds break and reform due to the librational and translational motion of water molecules. The intermittent correlation function C(t) in Eq. (11) gives the probability that a given hydrogen bond at time 0 is also present at time t, even if the bond breaks and reforms in the time in between. The continuous correlation function S(t) in Eq. (12) gives the probability that a given hydrogen bond at time 0 remains all the way until a time t. Here, a hydrogen bond is defined when the angle of O–H····O is larger than 150° and the distance between donor and acceptor is within 3.5 Å.^42^ We follow this definition to analyze the hydrogen bond lifetime

$$C(t)=\frac{\left\langle h(0)h(t)\right\rangle }{\left\langle h\right\rangle },$$ (11)

$$S(t)=\frac{\left\langle h(0)H(t)\right\rangle }{\left\langle h\right\rangle }.$$ (12)

The hydrogen bond relaxation time is defined as the time when the correlation function decays to 1/e.^43^ The relaxation time from S(t) ( $\tau_{HB}$) can be interpreted as the average lifetime of a hydrogen bond, while that from C(t) ( $\tau_{R}$) describes the structural relaxation of hydrogen bonds, which is relevant to the long-time translational and orientational diffusion [Fig. 6(b)]. We analyze the hydrogen bond between PEG and its hydration water, and find that the structural relaxation time $\tau_{R}$ is about 30.9 ns at 220 K. It is close to the extrapolated average relaxation time from QENS. It indicates that the dynamics of supercooled hydration water at ∼220 K is mainly from the startup of structural relaxation of hydrogen bonds between water and PEG, while $\tau_{HB}$ is nearly 7 ps at 220 K and no big difference with the increase in temperature (Table III).

**TABLE III.** Hydrogen bound lifetime at different temperature.

T/K	150	180	200	220	240	260	280	300
$\tau_{R}$/ns	⋯	⋯	⋯	30.9	4.50	1.28	0.26	0.06
$\tau_{HB}$/ps	16	9.5	7.3	7.0	5.2	4.3	3.4	2.6

**FIG. 7.**
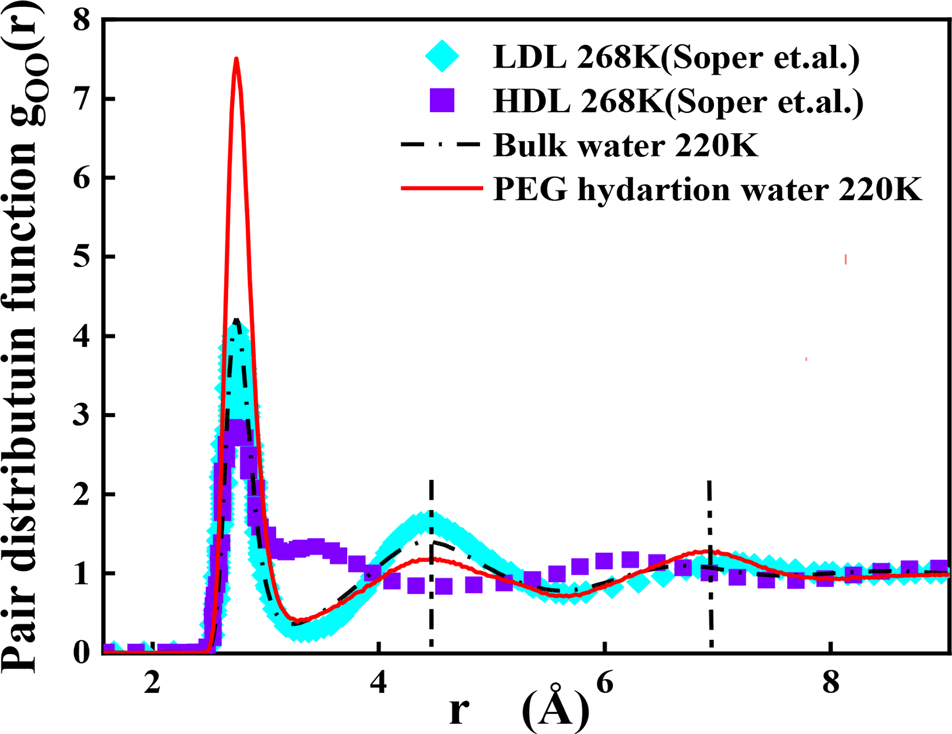
Oxygen–oxygen pair distribution functions of PEG hydration water, bulk water at 220 K. The LDL and HDL data at 268 K are from Soper *et al.*^44^

We compare the radial distribution function of water oxygen [ $g_{OO}(r)$] for bulk water and hydration water in Fig. 7. The experimental data of LDL and HDL from Soper and Ricci are used here for comparison.^44^ They found that the main differences between LDL and HDL are the second and third peak positions; that is, the former moves from 4.5 Å in LDL to 3.4 Å in HDL, and the latter shifts from 7.2 Å in LDL to 6.2 Å in HDL. In our system, the first and second peak positions of hydration water, bulk water, and LDL are almost the same at low temperature. The divergence of the relative amplitudes of the first peak from hydration water to bulk water is caused by the limited number of hydration water around the PEG backbone. It is probably from the excluded volume effect from the solute and does not necessarily impact strong interaction. Moreover, the third peak position of hydration water shifts to the right, from 6.7 Å (bulk water) to 6.9 Å, which is very similar to LDL. Thus, considering that the most bond ordered structure of water is the ice crystal,^45^ the hydration water at 220 K has a more bond ordered structure than bulk water.^46^

**FIG. 8.**
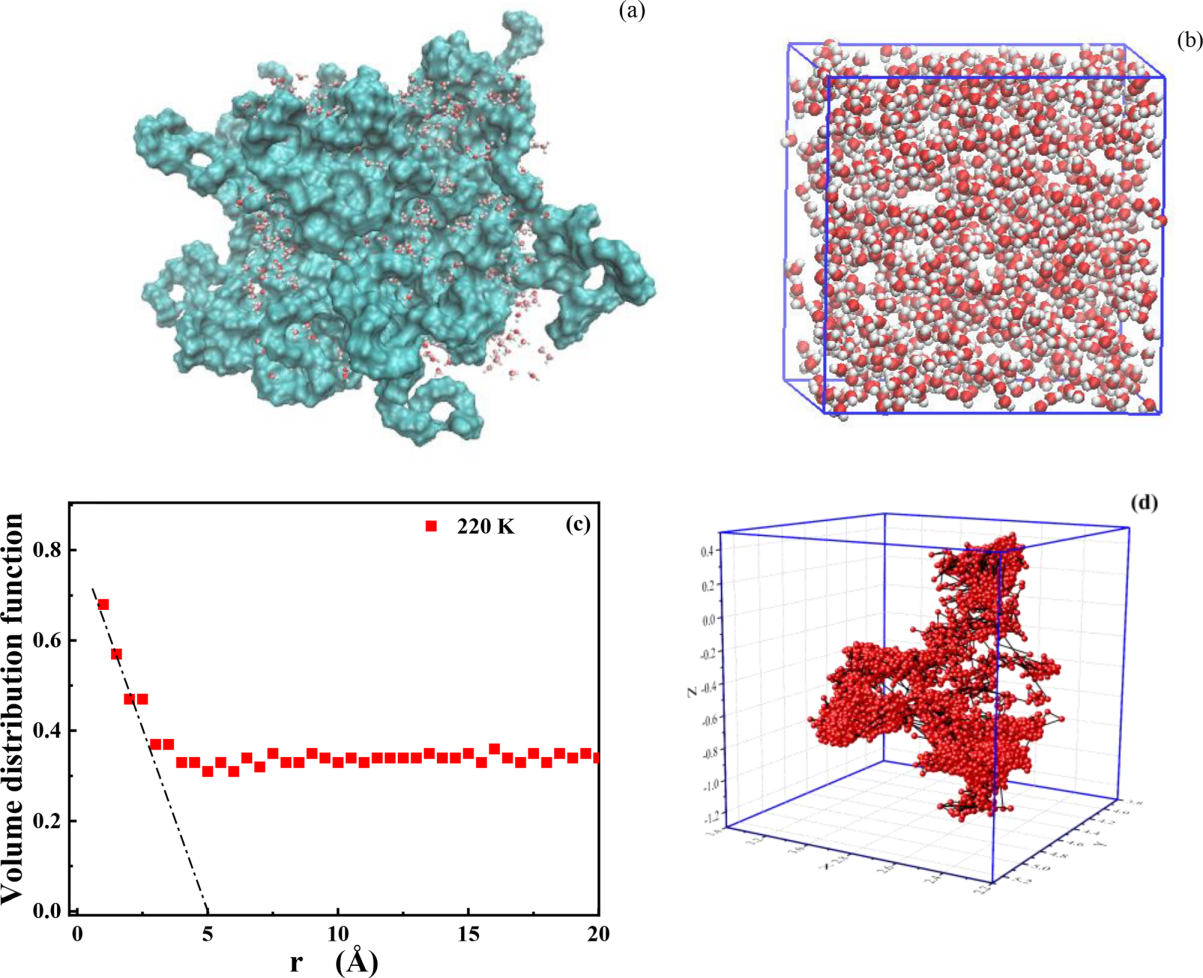
(a) Distribution of hydration water on the surface of PEG. The red and white ball-and-stick model represents water molecules, and the blue surface model represents PEG. (b) The simulation box of 30 wt. % water in PEG solution at 220 K showing water only. (c) Water volume distribution function of 30 wt. % water in PEG solution at 220 K. The slope from r = 0 across to the horizontal axis at r = 5 Å indicates the “confining length” of water. The small oscillation at small r arises from the finite width of bin to estimate this function. (d) Trajectory of one water molecule in PEG hydration shell (within 3.5 Å) at 220 K for 500 ns with a time step of 20 ps. The unit of coordinate is nm.

Hydration water around the PEG surface at 220 K can be schematically shown in Fig. 8. Water molecules are isolated into pools on the PEG surface [Fig. 8(a)], while in the interior [Fig. 8(b)], it exhibits a water-filled structure in the PEG matrix. The structure of those percolated water can be described by a volume distribution function.^47^ Figure 8(c) is the volume distribution function obtained from 30 wt. % water in PEG solution at 220 K. The slope from r = 0 across to the horizontal axis at r = 5 Å indicates the “confining length” of water, which is similar with the results from other soft confinement.^48^ The asymptotic behavior at large r of these functions represents the volume fraction of the water in the solution, which is about 35% at this concentration. Note that this distance cannot be interpreted as implying the clusters are necessarily spherical in shape and just represents lateral extent of water network. The trajectory from the hydration water molecules (within 3.5 Å of the main chain oxygen atoms, traced one) is show in Fig. 8(d). Under the same time step, it looks like water has performed a randomly short-range librational motion in one pool, and then diffused with a long-range to the next one. Therefore, the kinetic mechanism of this system can be explained by combining the QENS data. At supercooled temperature (10 K), all of their movements are frozen. As the temperature increases, the hydrogen atom begins to vibrate, and the temperature-dependent elastic intensity begins to decline. At ∼209 K, the break and re-form of the hydrogen bonds at low energy range begins, water molecules inside each pool can relax. When it increases to ∼220 K, water molecule inside a pool can jump to the adjacent ones with high energy. We suggest that the above sub-diffusion of hydration water should be consist with the water trapping sites diffusion hypothesis described by Singwi and Sjölander.^49^ At 220 K, the hydration water is trapped in in pool with librational motion, then translational diffuse to the next site.

To be clear, we have not analyzed the motion of PEG at supercooled temperature so far. Wood *et al.* have found that the motion of proteins with many side groups, such as maltose binding protein (MBP), is highly coupled with its hydration water.^50^ Note that PEG has no side group. We then assume that the high energy scale transition of PEG at 220 K is initiated by CH_2_ cranking motion. It is triggered by the structural relaxation of the hydrogen bond between water and PEG. The low energy scale transition of PEG at 234 K should be the onset of its structural relaxation.

## IV. CONCLUSION

To conclude, this research provides a microscopic image of the supercooled hydration water around PEG. Elastic scattering scanning shows that both PEG and water undergo a dynamic transition at supercooled temperature ranging from 200 to 240 K. The dynamics of PEG is coupled with its hydration water at supercooled temperature. The onset of hydrogen bond break and re-form between water and PEG happens at 209 K, followed by the translational jump diffusion of water at 220 K. The latter motion also facilities the CH_2_ cranking motion at PEG's backbone. Finally, the structural relaxation of PEG is activated at 234 K. Note that the transition temperature of hydration water increases from 209 to 220 K when reducing the instrument resolution from 1 to 17.5 *μ*eV. It proves that this transition is a dynamic process. The same is true for PEG. Due to the hydrophilic nature of PEG, water is separated into pools at PEG's hydrophilic sites on its surface. The motion of hydration water is a combination of in-cage libration and out-of-cage jump diffusion. They have highly bond ordered structures toward LDL at supercooled temperature and are the precursor of crystal ice.

## Data Availability

The data that support the findings of this study are available from the corresponding author upon reasonable request.
